# A System of Surgery

**Published:** 1872-09

**Authors:** 


### Books Reviewed.

---

*System of Surgery.* By Samuel D. Gross, M. D., LL. D., D. C. L. Oxon. Fifth Edition. In Two Volumes. Philadelphia: Henry C. Lea, 1872.

The System of Surgery by Gross has long been familiar to the medical public, both in our own and other countries, so that there is little occasion for any lengthy notice. There are changes in this addition which we take great pleasure in noticing. It has been enlarged both in the size and the number of its pages, and is illustrated by upwards of fourteen hundred engravings; nearly every subject is thus made plainer and more easily understood. New subjects are introduced, and the chapters generally extended. Ophthalmology of which comparatively little was said, receives in this edition the consideration which it deserves in a Surgery of such magnitude and scope, and tne general surgeon will find it complete so far as his wants, at least, require. The same may be said of Laryngoscopy and diseases of the throat; it is wholly adequate as a practical guide. Every other topic of special surgery has received consideration to a degree sufficient to make the work almost a complete guide in all departments.

This great American Surgery is justly a national pride, and its distinguished author a national honor. It is our American Standard, and Surgeons compare and estimate everything by it. We do not mean to say that every part of its teaching is truth, and that all else differing from it is error, or that it contains all that is known upon the various subjects of surgery, but it is certainly the most comprehensive, clear and satisfactory surgical guide within reach of the surgeon and student. The illustrations are excellent and well chosen—the text is clear and easily understood. It is extensive in its scope, and includes nearly every surgical subject; in a word, it is a complete System of Surgery.

We cannot omit in this connection to speak of the elegant and substantial style of its publication. It is matter for congratulation both to the author and profession that this valuable work is published in style to do honor to it, as well as the publishing house of Henry C. Lea.

